# Using texture analysis in the development of a potential radiomic signature for early identification of hepatic metastasis in colorectal cancer

**DOI:** 10.1016/j.ejro.2022.100415

**Published:** 2022-03-21

**Authors:** Laurence Devoto, Balaji Ganeshan, Deborah Keller, Ashley Groves, Raymond Endozo, Tan Arulampalam, Manish Chand

**Affiliations:** a Wellcome / EPSRC Centre, for Interventional and Surgical Sciences, University College London, 1st Floor, Charles Bell House, 43-45 Foley Street, London W1W 7TS, United Kingdom; b Institute of Nuclear Medicine, 5th floor, Tower, University College London Hospital, 235 Euston Road, London NW1 2BU, United Kingdom; c ICENI Centre, Colchester Hospital, Turner Rd, Mile End, Colchester CO4 5JL, United Kingdom

**Keywords:** Cancer, Metastases, Imaging, Radiology, Surgical management

## Abstract

**Background:**

Radiomics allows information not readily available to the naked eye to be extracted from high resolution imaging modalities such as CT. Identifying that a cancer has already metastasised at the time of presentation through a radiomic signature will affect the treatment pathway. The ability to recognise the existence of metastases earlier will have a significant impact on the survival outcomes.

**Aim:**

To create a novel radiomic signature using textural analysis in the evaluation of synchronous liver metastases in colorectal cancer.

**Methods:**

CT images at baseline and subsequent surveillance over a 5-year period of patients with colorectal cancer were processed using textural analysis software. Comparison was made between those patients who developed liver metastases and those that remained disease free to detect differences in the ‘texture’ of the liver.

**Results:**

A total of 24 patients were divided into two matched groups for comparison. Significant differences between the two groups scores when using the textural analysis programme were found on coarse filtration (p = 0.044). Patients that went on to develop metastases an average of 18 months after presentation had higher levels of hepatic heterogeneity on CT.

**Conclusion:**

This initial study demonstrates the potential of using a textural analysis programme to build a radiomic signature to predict the development of hepatic metastases in rectal cancer patients otherwise thought to have clear staging CT scans at time of presentation.

## 1 Introduction

Over the last quarter century, local recurrence rates for rectal cancer have improved with the introduction of neoadjuvant therapy, improvements in imaging and surgical technique, and multidisciplinary team care [1]. Despite this, a significant rate of rectal cancer patients will develop metastatic disease to the liver with an inherently worse prognosis [2]. Disease recurrence within 12 months of diagnosis is thought to be due to undetected metastatic disease at baseline. Current surveillance strategies to detect metastatic disease include serum CEA and CT imaging at variable intervals, which can often mean a delay to diagnosis. Immediate identification of occult and early hepatic metastasis is of critical importance to guide treatment and ultimately improve prognosis.

Radiomics is an emerging field in which important predictive information not immediately visible to the naked eye can be extracted using computer software and algorithms. Texture analysis (TA) is such an example of a novel imaging software that examines pixels on cross-sectional imaging and could add value to existing tumor markers [3]. TA evaluates heterogeneity or grey-level intensity variations within a ‘region of interest (ROI)’ on CT or MR imaging [4]. CT-TA has shown benefit for diagnosis, staging, nodal status, and response to therapy in multiple tumor types [5], [6], [7], [8]. In colorectal cancer, prior work has shown baseline and post-treatment variations in CT and MRI texture parameters correlated with the response to neoadjuvant therapy, primary disease recurrence, disease-free survival, and could be a predictive factor for overall survival [9], [10], [11]. Differences in CT texture after treatment have also been described valuable to assess the pathologic response to chemotherapy in patients with CRLM [3]. No work to date has used TA as a biomarker to stratify the pre-treatment scans of rectal cancer patients by susceptibility for development of liver metastases.

The aim of this pilot study was to create a novel radiomic signature using textural analysis in the evaluation of synchronous liver metastases in colorectal cancer. By examining the serial CT scans of patients during the course of treatment and the follow up surveillance period (5 years) to determine whether TA could identify early changes in those patients who subsequently developed liver metastases. Our hypothesis was that TA could detect early changes in liver texture indicating the development of metastases when compared to conventional high-resolution CT.

## 2 Materials and methods

### 2.1 Patient population and selection

**Fig. 1:**
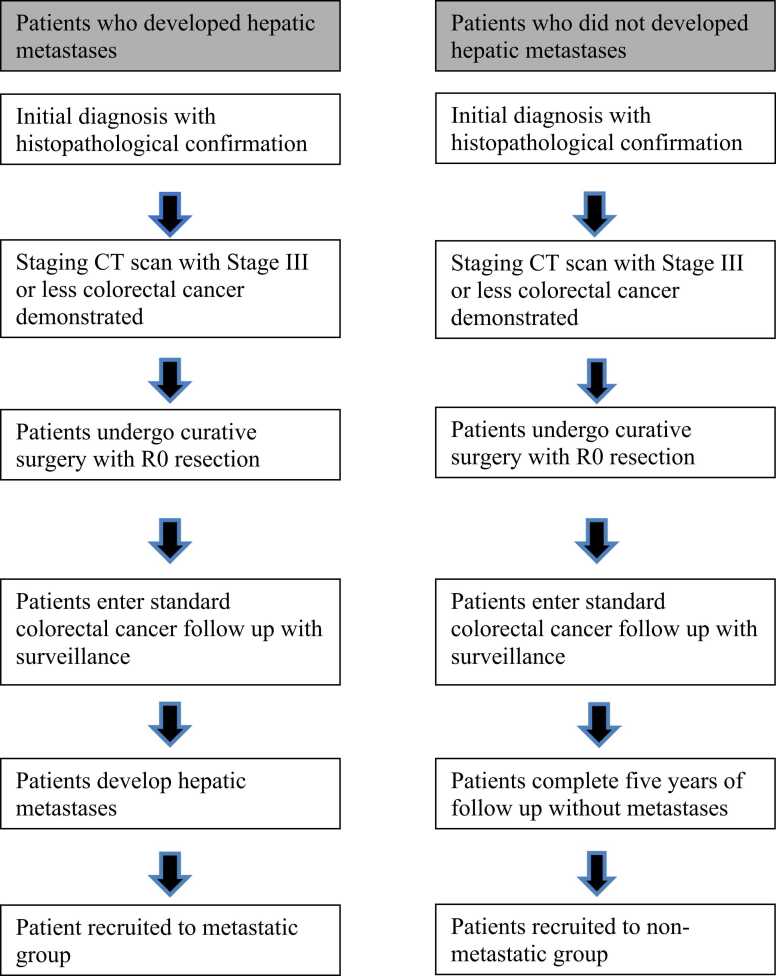
Patient selection flowchart.

The institutional cancer registry for a single tertiary referral center was reviewed. This is a prospectively kept and maintained database. The study group patients were eligible for analysis if they fulfilled all of the following criteria: histopathologically confirmed rectal adenocarcinoma; initial diagnosis with local disease only on T2 weighted MRI with no evidence of distant metastases on preoperative staging contrast enhanced CT scan (Stage III or less); underwent curative resection of the primary tumor demonstrating R0 resection and a mesorectal plane of excision; radiological/pathological evidence of the development of colorectal liver hepatic metastasis following surgery. The number and size of metastases was not specified in the criteria and any evidence of metastatic liver disease was included. Comparison was made with a matched control group with histopathologically confirmed rectal adenocarcinoma that underwent identical staging and treatment but did not develop subsequent colorectal liver hepatic metastasis diagnosed after surgery (Table 1)– this was confirmed by way of CT scan during the 5-year follow-up period. The primary surgery for both cohorts was performed between 2007 and 2012, allowing at least 5 years to monitor disease recurrence. Fig. 1. In addition, a review of each patients notes at the time of the study was conducted to assure that there was no current evidence of metastases.

**Table 1:** Patient Demographic Data.

	Metastasis	No Metastasis	p-value
Age (Mean SD)	68.75	64.5	0.4088
Gender (n, %)			
Male	5	9	0.285049
Female	7	2	0.095581
Approach			
Laparoscopic	5	4	0.738883
Open	7	8	0.796253
Pre-operative staging (MR)			
I	0	0	1
II	3	4	0.705457
III	5	6	0.763025
IV	1	0	0.317311
Post-operative pathological staging			
0	0	3	0.083265
I	0	1	0.317311
II	3	3	1
III	9	3	0.083265
IV	0	0	1
			
EMVI			
EMVI +ve	8	1	0.019631
EMVI - ve	4	9	0.165518

### 2.2 Texture analysis

The study and control groups had texture analysis performed on the staging, CT of the liver using the commercially available TexRAD research software. (TexRAD Ltd, Feedback Plc, Somerset, England, United Kingdom). Contrast enhanced images were used for analysis and comprised of a 10 mm section of mid liver obtained with a 1 s scan duration at a tube current of 300 mA and a tube voltage of 120 kVp (CT Seimens SOMATOM definition AS+ Slice thickness – 5 mm Filter – 31 F Field of view – 40 Portal phase.) The same CT scanner was used during the collection period of the staging CT scans. TexRAD uses software that highlights tumor heterogeneity features and uses histogram analysis to quantify and assess the distribution of grey-levels, coarseness and regularity within a lesion. It allows the heterogeneity parameters measured at different spatial scales to be compared and presented as ‘texture ratios’ or ‘texture spectra’ enabling quantitative assessment of imaging biomarkers within a suspected tissue. For study patients, the regions of interest (ROIs) on the CT scans underwent a filtration-histogram technique, where the image is extracted and enhanced along a texture spatial scale filter (SSF) scale corresponding to the ROI size. An ROI was delineated initially around the liver outline for the largest cross-sectional area. The ROI was automatically identified by the TA software to include the entire liver volume, then refined by the exclusion of areas of air with a thresholding procedure that removed any pixels with attenuation values below − 50 HU from the analysis. A range of parameters, including mean gray-level intensity, uniformity, entropy, mean of positive pixels, skewness, kurtosis, and standard deviation of the pixel distribution histogram were calculated without filtration and with Laplacian of Gaussian (LoG) spatial filter with various filter values for fine (2.0), medium (3.0, 4.0, 5.0), and coarse (6.0) textures. TA was also performed without the filtration step on the conventional CT in the control group for comparison. This methodology has been previously described for technique and clinical application [12], [13], [14], [15]. The current understanding of the pathophysiology is that micrometastases from the tumour that have entered into the hepatic circulation block portal flow. This causes localised changes where there is decreased portal venous distention increased hepatic arterial distention and changes to the density at a microscopic level with clumps of cells proliferating throughout the liver. This is then seen as differences in the homogeneity of the signal and hence grey-level produced by CT scan [16].

### 2.3 Outcomes measures

The main outcome measure was the ability of CT texture analysis to identify patients who would develop CRLM on the initial imaging studies. The secondary outcome measure was the time to development of liver metastases as confirmed on CT imaging from the time of initial diagnosis.

### 2.4 Statistical analysis

Statistical analysis was performed by using IBM SPSS Statistics for Macintosh, Version 23.0 (IBM Corporation, Armonk, NY) and MedCalc version 12.7.2 (MedCalc Software, Ostend, Belgium). Descriptive statistics were used to describe the patient demographic data. For the two diagnostic groups, medians (IQR) were calculated for each texture parameter on apparently normal appearing liver on CT without-filtration and filtered—fine, medium and coarse texture scales. The non-parametric two-tailed Mann Whitney test evaluated the difference between the two groups on the basis of their texture (without and with filtration) besides gender, age and body weight. Box and whisker plots (along with median and minimum/maximum values and quartiles) were generated to demonstrate differences (most-significant) in texture parameters on CT. For the best texture parameter diagnostic threshold to identify patients who developed liver metastases from those who did not develop liver metastases was identified based on area under the receiver-operating characteristics (ROC) analysis along with the sensitivity and specificity values. The ability of the best CT texture parameter to help predict survival (time to recurrence / CRLM) was assessed using Kaplan-Meier survival analysis. The threshold value used for survival analysis was established by using ROC analysis. Survival curves for patients above and below the threshold value were constructed to display the proportion who did not develop liver metastases at any given time. Differences between survival curves were evaluated using a non-parametric log rank test. For all statistical analyses, statistical significance was defined at alpha less than 0.05.

## 3 Results

A total of 24 patients were included with 12 patients in each group. The texture parameters for ‘without filtration’ and ‘filtered’ contrast-enhanced CT images for the two diagnostic groups are summarized in Appendix 1.

On review one of the patients in the non-metastatic group was found to have colonic adenocarcinoma incorrectly recorded in the database as rectal and so was excluded from the study.

**Fig. 2:**
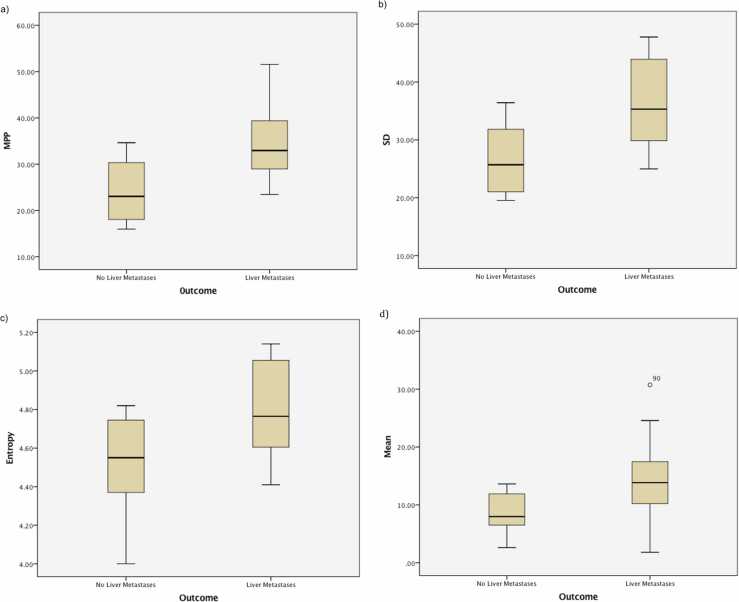
Coarse texture scale on contrast enhanced images was significantly higher in patients who developed liver metastases compared to patients who did not develop liver metastases. Box and whisker chart shows median, inter-quartile range and range for a) MPP (p = 0.007), b) SD (p = 0.013), c) Entropy (p = 0.032), d) Mean (p = 0.044).

There were no significant differences for the texture parameters between the two diagnostic groups for texture analysis without filtration. Filtered texture analysis of the whole, apparently normal appearing liver on the baseline CT was more heterogeneous and significantly different in patients who developed liver metastases compared to a more homogeneous texture quantification in those patients who did not develop liver metastases. Medium to coarse texture features quantified as mean intensity, standard-deviation, entropy and mean of positive pixels (MPP) were the markers of heterogeneity which were significantly higher in patients who developed liver metastases compared to patients who did not develop liver metastases. Particularly the texture difference was more amplified at the coarser filter scale (mean, p = 0.044; SD, p = 0.013; entropy, p = 0.032; MPP, p = 0.007, Fig. 2.) compared to medium filter scales (mean, p = 0.044; SD, p = 0.016; entropy, p = NS; MPP, p = 0.019).

**Fig. 3:**
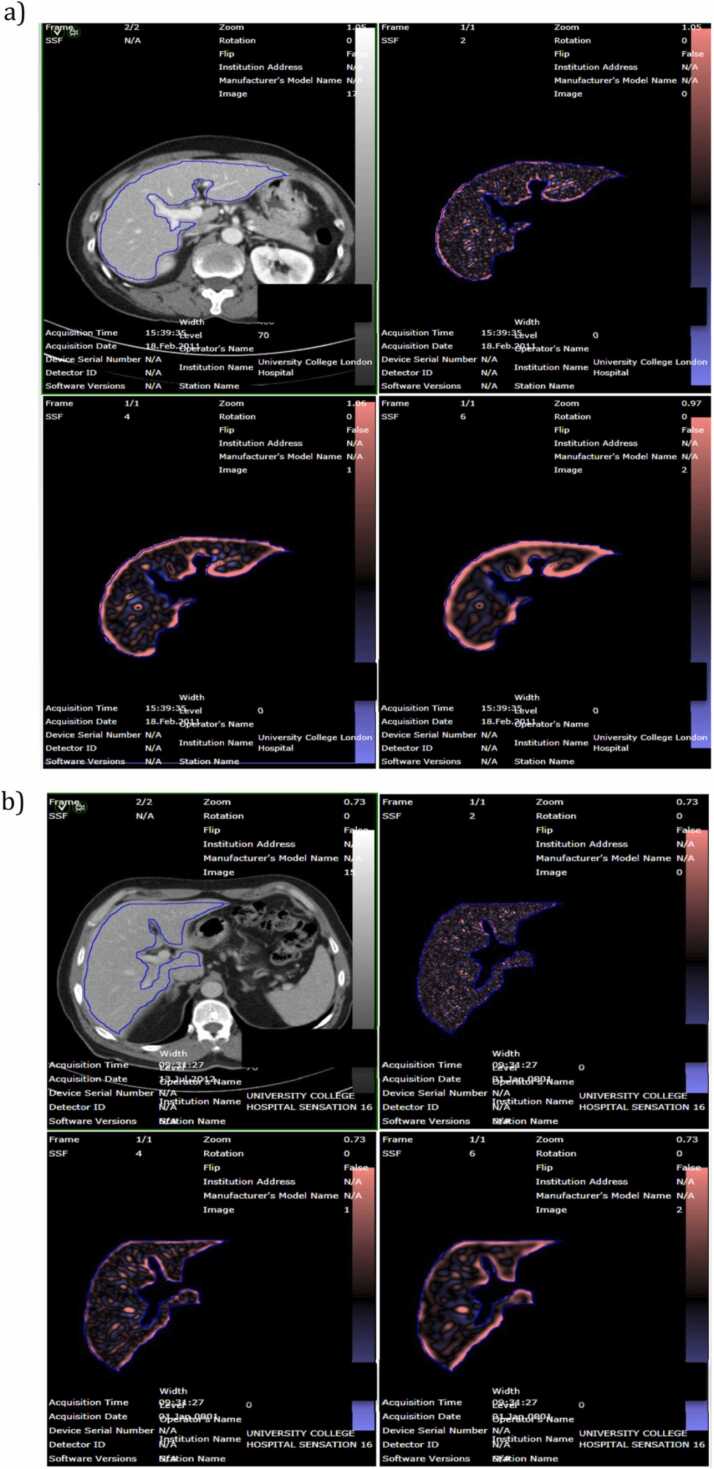
Illustration of the CTTA and MRTA as applied on two cases; a) one which developed and b) one which did not develop a liver metastasis.

A demonstration of the active region of interest with the texture analysis applied is shown in Fig. 3.

For a coarse filter scale, mean> =9.285 identified patients who developed liver metastases from patients who did not develop liver metastases (area under ROC curve = 0.750, p = 0.044, sensitivity = 83.3%, specificity = 63.6%). For a coarse filter scale, SD> =29.935 identified patients who developed liver metastases from patients who did not develop liver metastases (area under ROC curve = 0.803, p = 0.013, sensitivity = 75.0%, specificity = 63.6%). For a coarse filter scale, entropy> =4.605 identified patients who developed liver metastases from patients who did not develop liver metastases (area under ROC curve = 0.761, p = 0.032, sensitivity = 75.0%, specificity = 63.6%).

For a coarse filter scale, MPP> =27.8 identified patients who developed liver metastases from patients who did not develop liver metastases (area under ROC curve = 0.826, p = 0.007, sensitivity = 83.3%, specificity = 72.7%).

### 3.1 Survival-analysis

**Fig. 4:**
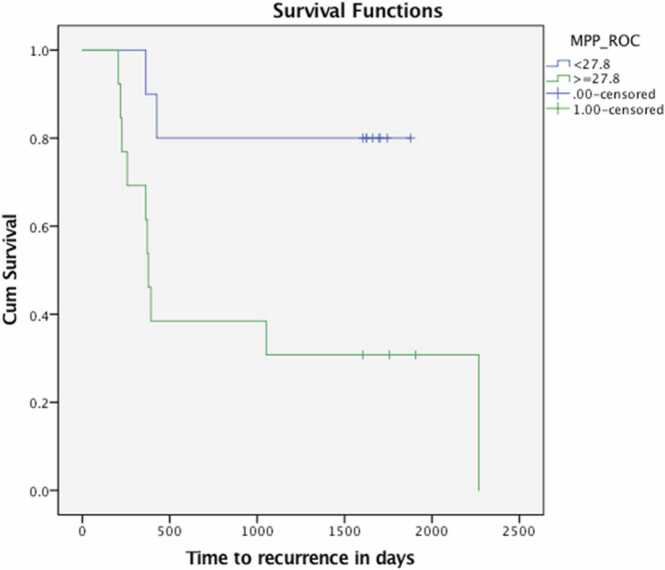
Kaplan-Meier survival curves for patients with apparently normal appearing liver on conventional contrast-enhanced CT separated by MPP. Survival curves were significantly different for MPP (p = 0.018).

The median time to survival (recurrence / CRLM) of the patients was 75.6 months. 12 of 23 patients developed liver metastases. The shortest time to development of CRLM was 6.9 months Kaplan-Meier survival curves were significantly different (p = 0.018) for MPP at the threshold value of > =27.8 (Fig. 4). Median survival in the poor prognostic group was 12.6 months.

## 4 Discussion

The results of the present study demonstrate that TA can potentially predict the development of liver metastases in patients about to undergo curative surgery for rectal cancer by identifying subtle changes on the CT scan. TA is most useful when the coarse filter is applied compared to the medium or fine filters. These changes are not immediately seen on conventional CT scanning used for staging in the United Kingdom. The timing of the analysis taking only a few hours allows for scalability to each new rectal cancer patient to be processed before their treatment is decided. The results can be interpreted easily with minimal training required for the medical team. These initial results demonstrate potential for a specific radiomic signature to detect hepatic metastases in colorectal cancer.

Currently, the risk of metastatic disease following curative resection for M0 rectal cancer is determined by the pathological staging of the tumour. Specific tumoural characteristics such as depth of penetration, extramural venous invasion and nodal involvement (stage III disease) mandate the use of adjuvant chemotherapy to mitigate the risk of distant disease [17]. However, despite the use of adjuvant therapy a significant proportion of patients still develop liver metastases. Surgery is the treatment of choice for RLM, but early detection is key [18]. In high-risk patients, metastases often develop within 12 months of surgical resection of the primary tumour – by definition this is considered synchronous disease. This simply means that those metastases are not detected at baseline and reflects the inadequacies of the current imaging modalities used for baseline staging to detect such synchronous metastases. TA provides an alternative and perhaps more accurate assessment to detect early distant disease within the emerging field of radiomics.

Surveillance strategies following rectal cancer resection are variable and there is no universal consensus on how best to manage patients. TA provides an additional surveillance strategy for specific patients considered at risk of developing metastatic disease. By performing TA on the surveillance CT scans, it may be possible to detect metastatic disease early and surgically resect or ablate. Optimising surveillance and managing distant disease is of paramount importance to improve survival outcomes. Determining which patients would be most at risk is another challenge. Currently, this is based on historical ‘bin models’ of staging using tumour depth and nodal disease (TNM staging) [19]. As we learn more about the biology of tumours, possible surveillance strategies would incorporate biological stratification with advanced analysis techniques such as TA. This pilot study opens the possibility of exciting future work. For example, future study would include biopsy to areas which have been identified by TA for pathological validation. This would determine whether early dysplastic or metastatic changes are actually occurring.

Ng et al. studied the CT features of primary colorectal cancer texture were studied in relation to 5-year overall survival rate. Tumours demonstrating less heterogeneity were associated with poorer survival, concluding that the addition of texture analysis to staging contrast-enhanced CT may improve prognostication in patients with primary colorectal cancer [9]. Similar results were found looking at response to treatment for to assess the pathologic response to chemotherapy in patients with CRLMs [3]. Lesion uniformity increased in the good responders while correspondingly entropy decreased. In the poor responders, the opposite effect was observed.

TA on the MRI scan of the primary tumor has been previously shown to be a marker of severity in terms of prognosis (OS, DFS, PFS) and treatment response (pathological-response) [11], [20] It is important to note that TA provides an analysis of the images and not necessarily a marker of biological activity or tumour aggression.

## 5 Limitations

Limitations of the study include the small sample used and the retrospective, non-randomized nature of the study. To better investigate the potential of TA a prospective study will be needed with greater numbers prior to external validation. Although the software is universal, prior to the analysis there is some degree of manual segmentation of the scans which is user-dependent. Furthermore, this study did not compare TA with MR liver or PET scanning. These more advanced imaging modalities have been shown to better detect changes in liver architecture and tumoral activity, however these modalities are not universally used in staging nor surveillance and further have huge cost implications. An interesting future work would be comparison to these modalities or even using TA on MR scans of the liver.

In addition, the small number of patients meant that multivariate logistic regression was not possible considering the number of variables examined. Secondly the study was retrospective allowing for the possibility of selection and confounding bias. Finally, the multiple variables assessed by the textural analysis program has the potential for false discovery however we are confident in the strong correlation found in the results and the numerical threshold required by the program to correctly be interpreted as indicating metastases. This pilot study has demonstrated a clear indication of the relationship between textural features and the development of metastases and a planned follow up study that is prospective with a lager cohort of patients will be able to fully interrogate the influence of mean intensity, standard-deviation, entropy and mean of positive pixels.

## 6 Conclusion

This initial pilot study supported that in normal appearing liver texture analysis of the baseline staging CT was more heterogeneous and significantly different in patients who developed liver metastases compared to patients who did not develop liver metastases. Patient that eventually developed RLM had higher texture-scores, while those who did not develop RLM had a more homogeneous liver, with lower texture-scores. TA detects and measures ‘tumour complexity’ in CT images and reveals more information than is readily visible to the naked eye. The differences in heterogeneity data could potentially be used for more specific risk stratification, prognosis and management for rectal cancer patients that will develop liver metastasis. With the promising results of this pilot, further prospective validation is necessary to determine the power of TA in this application.
