# Supplementary material for: Using texture analysis in the development of a potential radiomic signature for early identification of hepatic metastasis in colorectal cancer
Source: Eur J Radiol Open. 2022 Mar 21;9:100415. doi: 10.1016/j.ejro.2022.100415 (PMC8942820; doi:10.1016/j.ejro.2022.100415)
Supplement: Supplementary file 1 — Supplementary material [file mmc1.docx]

Supplementary Files

Appendix 1 CT Texture Analysis of Patients Who Did and Did Not Develop Hepatic Metastasis

| Characteristic Data | Metastasis | No Metastasis | p-value |
| --- | --- | --- | --- |
| No filtration |  |  |  |
| TX_sigma |  |  |  |
| mean | 95.68 | 92.29 | 0.091 |
| Standard Deviation | 14.64 | 14.29 | 0.740 |
| Entropy | 4.055 | 4.06 | 0.880 |
| MPP | 95.68 | 92.29 | 0.091 |
| Skewness | 0.41 | 0.27 | 0.211 |
| Kurtosis | 0.99 | 0.76 | 0.169 |
| Filter 2.0 (fine) |  |  | 0.211 |
| TX_sigma |  |  |  |
| mean | 1.31 | 0.74 | 0.235 |
| Standard Deviation | 33.57 | 29.29 | 0.091 |
| Entropy | 4.89 | 4.78 | 0.134 |
| MPP | 27.08 | 23.43 | 0.091 |
| Skewness | 0.62 | 0.29 | 0.118 |
| Kurtosis | 1.72 | 0.95 | 0.288 |
| Filter 3.0 (med) |  |  |  |
| TX_sigma |  |  |  |
| mean | 3.58 | 2.06 | 0.091 |
| Standard Deviation | 30.04 | 25.59 | 0.069 |
| Entropy | 4.71 | 4.58 | 0.059 |
| MPP | 24.63 | 21.57 | **0.023** |
| Skewness | 1.19 | 1.08 | 0.413 |
| Kurtosis | 3.27 | 3.19 | 1.000 |
| Filter 4.0 (med) |  |  |  |
| TX_sigma |  |  |  |
| mean | 6.86 | 4.04 | 0.051 |
| Standard Deviation | 30.37 | 24.17 | **0.032** |
| Entropy | 4.66 | 4.51 | 0.059 |
| MPP | 26.07 | 19.99 | **0.044** |
| Skewness | 1.27 | 1.39 | 0.880 |
| Kurtosis | 3.18 | 3.70 | 0.379 |
| Filter 5.0 (med) |  |  |  |
| TX_sigma |  |  |  |
| mean | 10.35 | 6.13 | **0.044** |
| Standard Deviation | 32.78 | 24.81 | **0.016** |
| Entropy | 4.71 | 4.52 | 0.069 |
| MPP | 29.47 | 21.66 | **0.019** |
| Skewness | 1.18 | 1.34 | 0.740 |
| Kurtosis | 2.63 | 3.56 | 0.134 |
| Filter 6.0 (coarse) |  |  |  |
| TX_sigma |  |  |  |
| mean | 13.85 | 7.98 | **0.044** |
| Standard Deviation | 35.31 | 25.71 | **0.013** |
| Entropy | 4.77 | 4.55 | **0.032** |
| MPP | 32.96 | 23.04 | **0.007** |
| Skewness | 1.09 | 1.20 | 0.880 |
| Kurtosis | 2.31 | 2.27 | 0.413 |

**References**
